# Improved method for measurement of multi-walled carbon nanotubes in rat lung

**DOI:** 10.1186/s12995-016-0132-7

**Published:** 2016-09-15

**Authors:** Makoto Ohnishi, Masaaki Suzuki, Masahiro Yamamoto, Tatsuya Kasai, Hirokazu Kano, Hideki Senoh, Ichiro Higashikubo, Akihiro Araki, Shoji Fukushima

**Affiliations:** 1 Japan Bioassay Research Center, Japan Organization of Occupational Health and Safety, 2445 Hirasawa, Hadano, Kanagawa 257-0015 Japan; 2 Occupational Health Research and Development Center, Japan Industrial Safety and Health Association, 5-35-1 Shiba, Minato-ku, Tokyo, 108-0014 Japan

**Keywords:** Multi-walled carbon nanotubes, Rats, Lungs, Carbon black, Benzo[ghi]perylene, Intratracheally, HPLC, Quantitation, Improved method, Validation

## Abstract

**Background:**

Previously, we have developed and reported the method of measuring multi-walled carbon nanotube (MWCNT) in the lung from rats exposed to MWCNT intratracheally. The present research was performed to improve the analytical method of MWCNT to measure multiple samples in a short period of time. For the xanalysis of MWCNTs from tissues, the existence of carbon black may interfere. Therefore, it was examined whether or not carbon black interfere the determination of MWCNT in the standard solutions. Then, MWCNTs were administered to rats and the MWCNTs were determined in the rats by the new method and the recovery rates and time for determination were calculated. The standard solutions for MWCNTs and carbon black were prepared, and the concentrations in the solutions were determined by HPLC with checking their linearity between the concentrations and signal intensities. The reproducibility of the determination was also checked.

**Methods:**

The concentrations of MWCMTs in the standard solutions were determined by HPLC with a fluorescent detector. Those of carbon black were also determined using the same method. The MWCNTs were administered to rats intratracheally. The MWCNTs in the lung were determined in a newly modified method including digestion of lung tissues by strong alkali solution and marking MWCNTs by benzo[*ghi*]perylene. The time for the determinations was recorded and the recovery rate of MWVNTs was calculated.

**Results:**

MWCNT showed linearity in a range of 0.2 to 1.0 μg/mL. In contrast, carbon black demonstrated a very low slope, showing flat pattern. Regarding the reproducibility of the analysis, the coefficient of variation was lower than 10 %. The analysis of 20 samples were completed in 1.5 h. The recovery rates of MWCNT from the lung of rats receiving intratracheal MWCNT administration were 101 to 102 %.

**Conclusions:**

The improved method for measuring MWCNT allows an efficient MWCNT quantitation in a short period of time. Also, a small amount of MWCNTs can be measured without influence of carbon black.

## Background

Recently, we have developed the analytical method of multi-walled carbon nanotube (MWCNT) measurement in the rat lung and reported the results obtained in the experiments with MWCNT inhalation exposure and intratracheal administration in rats [1]. This method was also applied in rats which were subjected to 2-and 13-weeks MWCNT exposure by inhalation [2, 3]. By the method, the inter-animal variation was big and it took 2 h for the determination of 20 samples, which is a relatively long period of time. Therefore, we developed a new method that uses benzo[*ghi*]perylene (B(ghi)P) as a marker which attached with MWCNTs. For the analysis of MWCNTs from tissues, the existence of carbon black may interfere. Therefore, it was examined whether or not carbon black interfere the determination of MWCNT in the standard solutions in a new method. Then, MWCNTs were administered to rats and the MWCNTs were determined in the rats by the new method and the recovery rates and time for determination were calculated.

## Methods

### Test substance

The MWCNT with an average width of 90.7 nm and length of 5.7 μm was purchased from Hodogaya Chemical Co. Ltd. (MWNT-7, Lot No. 080126, Tokyo, Japan). Carbon black (CB) (JIS Test Powders 1 Class 12, Lot No. 2KA21) was purchased from The Association of Powder Process Industry and Engineering (Kyoto, Japan), and the particle diameter ranges were 30–200 nm.

These substances were used in the present study as produced; i.e., without being purified or further sieved. Since MWCNTs and CB are not water soluble, the test substances were suspended in Clean99 K-200 (C99) as a colloidal dispersant and subjected to ultrasonication for 30 min with an ultrasonic homogenizer (VP-30S, 20 kHz, 300 W, Taitec Co., Ltd., Tokyo, Japan).

### Calibration curve

A 10-mg sample of MWCNT was added to 40 mL of the TW-mixture and sonicated for 30 min. The solution was diluted to 1 μg/mL with C99. This solution was used to prepare additional standards of 0.2, 0.4, 0.6 and 0.8 μg/mL MWCNT in C99. The prepared solutions were handled similarly “A newly developed method for digestion of lung tissue and determination of MWCNTs” following.

### Animals and treatment

Male F344/DuCrlCrlj rats were purchased from Charles River Japan, Inc. (Kanagawa, Japan) at the age of 12 weeks. The animals were quarantined, acclimated for 1 week, and then housed individually under controlled environmental conditions. The room temperature in the animal room was controlled at 23 °C ± 2 °C and the relative humidity at 55 % ± 15 % with 15 to 17 air changes/hour. Fluorescent lighting was controlled automatically to provide a 12-h light/dark cycle. All rats had free access to sterilized water and γ-irradiation-sterilized commercial pellet diet (CRF-1, Oriental Yeast Co., Ltd., Tokyo, Japan). This study was approved by the Animal Experiment Committee of the Japan Bioassay Research Center. Animal care was in accordance with the Guidelines for Proper Conduct of Animal Experiments (Science Council of Japan, 2006).

Before the intratracheal administration, the ultrasonicated suspension of MWCNT was further subjected to additional ultrasonication for 30 s with a sonicator (US-2, AS ONE Co., Ltd., Tokyo, Japan). Animals were 5 rats per group (total 10 rats) were used for the intratracheal administration of MWCNT. Rats received MWCNTs at a dose of 60 (Group A) and 120 μg/animal (Group B). When starting the administration, the mean body weights and standard deviations in Groups A and B were 259 ± 10 g (5 rats) and 259 ± 11 g (5 rats), respectively. After the inhalational anesthetization with the isoflurane gas (Forane, Abbott Japan Co., Ltd., Tokyo, Japan), the 0.3 ml of suspension of MWCNT in 9.6 % phosphate-buffered saline containing 0.1 % Tween 80 (TW-solution) was intratracheally administered to rats using a microspray cannula of an Intratracheal Aerosolizer (IA-1B, PennCentury, Inc., USA). Rats were euthanized, and the lungs and trachea were removed after 4 h of MWCNT administration, weighed, and fixed in 10 % neutral buffered formalin for 4 weeks. Then rat lungs were assessed for MWCNTs quantities by the method described below.

### A newly developed method for digestion of lung tissue and determination of MWCNTs

**Fig. 1:**
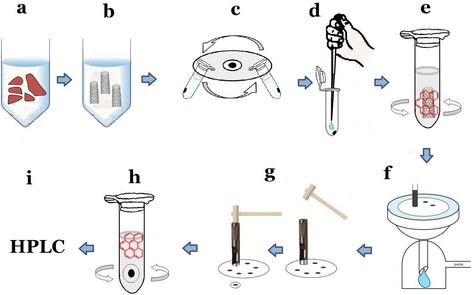
A newly developed for MWCNT. **a** Lung tissue with the C99 at room temperature. **b** Lung digestion in C99. **c** Centrifugation of lung solution at 12,000 rpm for 10 min. **d** Supernatant removal and 60 % sulfuric acid addition to MWCNT samples. **e** MWCNT-marker hybrid method: Marker adsorbtion onto MWCNT for 15 min. **f** MWCNTs adhering to Nuclepore membrane filters were extracted with marker on MWCNTs. **g** Filter with MWCNT was then punched out in circular disks **h** Addition of 1 ml acetonitrile, stirring, followed by the extraction of the marker in MWCNTs and the acetonitrile filtration of marker in the MWCNTs. **i** HPLC analysis

The lung samples were incubated with C99 at room temperature overnight and were digested (Fig. 1a and b) [4]. The digested solution of lungs by strong alkaline was then centrifuged at 12,000 rpm for 10 min (Fig. 1c). The supernatants were discarded and thereafter a 1 ml of TW solution was added followed by stirring and further centrifugation at 12,000 rpm for 10 min. Next, the supernatants were discarded again and 200 μl of concentrated sulfuric acid was added to the precipitate (Fig. 1d). The resultant solution was stirred and then, a 1 ml of TW-solution was added and samples were sonicated during a 10-s period. After adding 0.125 μg/ml B(ghi)P and 50 μl acetonitrile, the solutions were stirred for 15 min (Fig. 1e) to produce MWCNT samples with actual concentrations of 0.2, 0.4, 0.6, 0.8, and 1.0 μg/ml. Next, the MWCNTs adhering to Nuclepore membrane filters (Whatman; 111109, pore size; 0.8 μm, diameter; 47 mm) was performed (Fig. 1f). The filter with MWCNTs was then punched out in circular disks with a 8 mm- diameter (Fig. 1g). The disc was soaked in 1 ml of acetonitrile, and stirring. The attached B(ghi)P with MWCNTs was extracted by acetonitrile (Fig. 1h) and filtered before subjecting to the HPLC analysis (Fig. 1i).

Chromatography was performed using an Acquity UPLC system (Waters, Milford, MA, U.S.A.) coupled to a fluorescence detector FLR (Waters). Eluates were analyzed quantitatively by monitoring at fluorescent wavelengths of 294 nm for excitation and 410 nm for emission with 5 μl aliquots of extract injected onto a 1.7 μm C18 100 × 2.1 mm I.D. Acquity BEH column (Waters) (Fig. 1i). The mobile phases were acetonitrile : methanol : distilled water =75 : 20 : 5. The peak of the injected sample was detected at about 1.3 min. An eluent flow rate of 0.5 ml/min was used for all analyses.

### The improved method was estimated to perform the following three validations of calibration curve, reproducibility and recovery

#### Validation 1. Calibration curve of MWCNT and CB

Using the improved method, 0.2, 0.4, 0.6, 0.8, and 1.0 μg/mL samples of MWCNT and CB were measured, and the linearity was assessed.

#### Validation 2. Reproducibility of MWCNT

Twenty samples each of MWCNT at concentrations of 0.2 and 1.0 μg/mL were measured by the improved method, and reproducibility was assessed.

#### Validation 3. Recovery of MWCNT administration in rat lung

MWCNT was intratracheally administered to rats (Group A and Group B). Immediately after administration, rats were subjected to autopsy. MWCNTs in the lung were measured, and the dose and the volume of MWCNTs were compared to assess the recovery rate.

## Results

### Calibration curve of MWCNT and CB

**Fig. 2:**
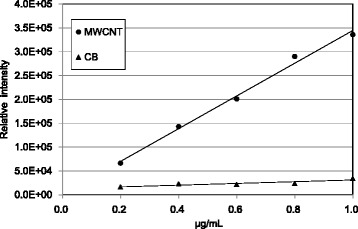
Calibration curves of MWCNT and CB

The calibration curves of MWCNT and CB are demonstrated in Fig. 2. A positive correlation was found between the MWCNT concentration (0.2–1.0 μg/mL) and the area value, showing a similar sensitivity to that of our previous method [1]. The correlation coefficient and p value were 0.9964 and 0.0000348. However, CB had a very low concentration-area slope, showing flat pattern, and all relative intensity values determined by HPLC were lower than the concentration of 0.2 μg/mL.

### Reproducibility of MWCNT

Among the calibration curve samples, the concentrations of 20 samples each for 0.2 and 1.0 μg/mL were determined, and the results are shown in Table 1. The measurement results were almost comparable to the established solution concentrations, and the coefficient of variation was less than 10 %, showing an acceptable reproducibility for MWCNT analysis. The analysis of 20 samples completed in 1.5 h by reducing the number of centrifugations from 4 to 2.

**Table 1:** Reproducibility of MWCNT using improve method

MWCNT	No. of samples	Measurement value (μg/mL)	SD (μg/mL)	C.V.(%)
0.2 μg/mL	20	0.193	0.017	8.7
1.0 μg/mL	20	0.995	0.069	6.9

### Recovery of MWCNT administration in rat lung

Immediately after the intratracheal MWCNT administration to rats, the deposition amount in the lung was measured. The results are shown in Table 2. While slight fluctuations were seen among the 5 rats, quantity of intratracheal administration of MWCNT and the quantities of MWCNT in the lung (including the trachea) were almost comparable.

**Table 2:** MWCNT measurements in the lung by intratracheal administration

MWCNT	No. of samples	Measurement value (μg/lung)	SD (μg/lung)	Recovery (%)
60 μg	5	60.4	15.9	101
120 μg	5	122.3	36.3	102

## Discussion

We developed the new method for analysis of very small amount of MWCNT in lung [1]. In this report, we investigated the effect of carbon black and the shortening of the analysis time for the MWCNT. These evaluation of the calibration curve revealed that selective measurement of MWCNT is possible even in CB coexistence conditions. The currently reported analytical procedures of MWCNT, such as carbon analysis [5–7] and spectrophotometry [8], have difficulties in the distribution between MWCNT and CB. This improved method is the only one able to differentiate MWCNT from CB.

Thorough diffusion of MWCNTs in the solution using ultrasonic homogenizer immediately before adding the marker, B(ghi)P, was important to suppress fluctuations. Furthermore, the time required for the analysis was shorter than 2 h by the previous method [1], showing that the analysis can be completed in a shorter period of time, which will lead to an increase in the number of samples assayed in a certain period of time.

These findings indicated that the present improved method to measure the MWCNT can be applied for the quantitation of its deposition in the lung.

## Conclusions

The present improved method of MWCNT measurement has the following benefits.

1. The improved method offers more efficiency as compared to the previous one in case of MWCNT quantitation in a short period of time (1.5 h for 20 samples).
2. Small amounts of MWCNTs could be measured without the influence of CB.

## Abbreviations

B(ghi)P: Benzo[*ghi*]perylene
C99: Clean99 K-200
CB: carbon black
HPLC: high performance liquid chromatography
MWCNT: multi-walled carbon nanotubes
SD: standard deviation
TW-solution: 9.6 % phosphate-buffered saline containing 0.1 % Tween 80

## References

[1] Ohnishi M, Yajima H, Kasai T, Umeda Y, Yamamoto M, Yamamoto S, Okuda H, Suzuki M, Nishizawa T, Fukushima S (2013). Novel method using hybrid markers: development of an approach for pulmonary measurement of multi-walled carbon nanotubes. J Occup Med Toxicol.

[2] Umeda Y, Kasai T, Saito M, Kondo H, Toya T, Aiso S, Okuda H, Nishizawa T, Fukushima S (2013). Two-week toxicity of multi-walled carbon nanotubes by whole-body inhalation exposure in rats. J Toxicol Pathol.

[3] Kasai T, Umeda Y, Ohnishi M, Kondo H, Takeuchi T, Aiso S, Nishizawa T, Matsumoto M, Fukushima S (2015). Thirteen-week study of toxicity of fiber-like multi-walled carbon nanotubes with whole-body inhalation exposure in rats. Nanotoxicology.

[4] Kohyama N, Suzuki Y (1991). Analysis of asbestos fibers in lung parenchyma, pleural plaques, and mesothelioma tissues of North American insulation workers. Ann NY Acad Sci.

[5] Ono-Ogasawara M, Myojo T (2011). A proposal of method for evaluating airborne MWCNT concentration. Ind Health.

[6] Tamura M, Inada M, Nakazato T, Yamamoto K, Endo S, Uchida K, Horie M, Fukui H, Iwahashi H, Kobayashi N, Morimoto Y, Tao H (2011). A determination method of pristine multiwall carbon nanotubes in rat lungs after intratracheal instillation exposure by combustive oxidationnondispersive infrared analysis. Talanta.

[7] Doudrick K, Corson N, Oberdörster G, Eder A, Herckes P, Halden R, Westerhoff P (2013). Extraction and quantification of carbon nanotubes in biological matrices with application to rat lung tissue. ACS Nano.

[8] Mercer R, Scabilloni J, Hubbs A, Battelli L, McKinney W, Friend S, Wolfarth M, Andrew M, Castranova V, Porter D (2013). Distribution and fibrotic response following inhalation exposure to multi-walled carbon nanotubes. Part Fibre Toxicol.

